# The Global DNA Methylation Surrogate LINE-1 Methylation Is Correlated with *MGMT* Promoter Methylation and Is a Better Prognostic Factor for Glioma

**DOI:** 10.1371/journal.pone.0023332

**Published:** 2011-08-04

**Authors:** Fumiharu Ohka, Atsushi Natsume, Kazuya Motomura, Yugo Kishida, Yutaka Kondo, Tatsuya Abe, Yoko Nakasu, Hiroki Namba, Kenji Wakai, Takashi Fukui, Hiroyuki Momota, Kenichiro Iwami, Sayano Kinjo, Maki Ito, Masazumi Fujii, Toshihiko Wakabayashi

**Affiliations:** 1 Department of Neurosurgery, Nagoya University School of Medicine, Nagoya, Japan; 2 Division of Molecular Oncology, Aichi Cancer Center Research Institute, Nagoya, Japan; 3 Department of Neurosurgery, Oita University School of Medicine, Oita, Japan; 4 Department of Neurosurgery, Shizuoka Cancer Center, Shizuoka, Japan; 5 Department of Neurosurgery, Hamamatsu Medical University, Hamamatsu, Japan; 6 Department of Preventive Medicine, Nagoya University School of Medicine, Nagoya, Japan; 7 FALCO biosystems, Kyoto, Japan; The University of Chicago, United States of America

## Abstract

Gliomas are the most frequently occurring primary brain tumor in the central nervous system of adults. Glioblastoma multiformes (GBMs, WHO grade 4) have a dismal prognosis despite the use of the alkylating agent, temozolomide (TMZ), and even low grade gliomas (LGGs, WHO grade 2) eventually transform to malignant secondary GBMs. Although GBM patients benefit from promoter hypermethylation of the *O* ^6^-methylguanine-DNA methyltransferase (*MGMT*) that is the main determinant of resistance to TMZ, recent studies suggested that *MGMT* promoter methylation is of prognostic as well as predictive significance for the efficacy of TMZ. Glioma-CpG island methylator phenotype (G-CIMP) in the global genome was shown to be a significant predictor of improved survival in patients with GBM. Collectively, we hypothesized that *MGMT* promoter methylation might reflect global DNA methylation. Additionally in LGGs, the significance of *MGMT* promoter methylation is still undetermined. In the current study, we aimed to determine the correlation between clinical, genetic, and epigenetic profiles including LINE-1 and different cancer-related genes and the clinical outcome in newly diagnosed 57 LGG and 54 GBM patients. Here, we demonstrated that (1) *IDH1/2* mutation is closely correlated with *MGMT* promoter methylation and *1p/19q* codeletion in LGGs, (2) LINE-1 methylation levels in primary and secondary GBMs are lower than those in LGGs and normal brain tissues, (3) LINE-1 methylation is proportional to *MGMT* promoter methylation in gliomas, and (4) higher LINE-1 methylation is a favorable prognostic factor in primary GBMs, even compared to *MGMT* promoter methylation. As a global DNA methylation marker, LINE-1 may be a promising marker in gliomas.

## Introduction

Glioblastoma multiforme (GBM, WHO grade 4) is one of the most frequently occurring brain tumors in the primary central nervous system of adults and is highly malignant. The median survival time is 14 months from diagnosis, despite the use of aggressive treatment, surgery, postoperative radiotherapy, and adjuvant temozolomide (TMZ)-based chemotherapy [1], [2], [3]. The efficacy of TMZ for treating GBM is often very limited because of inherent or acquired resistance. The main determinant of resistance to alkylating agents is *O* ^6^-methylguanine-DNA methyltransferase (MGMT); this enzyme directly and specifically eliminates the cytotoxic alkyl adducts formed at the *O* ^6^ position of guanine and less frequently at the *O* ^4^ position of thymine [4], [5], [6]. A subanalysis in an international randomized trial by the European Organization for Research and Treatment of Cancer/National Cancer Institute of Canada (EORTC/NCIC) compared the results of radiotherapy alone with those of concomitant radiotherapy and TMZ and showed that epigenetic silencing of the *MGMT* gene by promoter methylation increased the survival time of patients with primary GBM [3], [7]. MGMT has been used as a therapeutic target because downregulation of MGMT may enhance the chemosensitivity of malignant gliomas to TMZ. Thus, MGMT has been regarded as a predictive factor in the treatment of GBM patients. Although the predictive value of *MGMT* methylation has largely been confirmed in numerous prospective and retrospective clinical investigations, it is unclear if this is directly due to reduced MGMT expression. Indeed, evidence has shown that *MGMT* promoter hypermethylation is better correlated with survival benefit than evaluations of its mRNA and protein levels [8], [9]. In addition, Van den Vent et al reported that a methylated *MGMT* promoter was of prognostic significance among patients with anaplastic gliomas treated with radiation alone [10]. These results suggest that a methylated *MGMT* promoter is prognostic as well as predictive for the outcome of adjuvant therapy in high-grade gliomas [11].

Cancer-specific DNA methylation changes are hallmarks of human cancers, with global DNA hypomethylation often seen concomitantly with hypermethylation of CpG islands [12]. A CpG island methylator phenotype (CIMP) is regarded as cancer-specific CpG island hypermethylation of a subset of genes in some tumors [13]. Colorectal CIMP is associated with microsatellite instability and transcriptional silencing [14]. Recently, The Cancer Genome Atlas (TCGA) project and other groups have attempted to profile GBM genes comprehensively based on genomic and epigenomic aberrations and transcriptomal features [1], [15], [16]. In GBM, glioma-CIMP status (G-CIMP) was shown to be a significant predictor of improved patient survival [16]. Collectively, these different sets of observations suggest that the level of *MGMT* promoter methylation, serving as a prognostic factor, may reflect an aspect of the global DNA methylation status in GBM.

Recently, long interspersed nuclear element-1 (LINE-1) has attracted attention. LINE-1 is a non-long terminal-repeat class of retroposons that is the most successfully integrated mobile element in the human genome and accounts for approximately 18% of the human genome [17]. The level of LINE–1 methylation is regarded as a surrogate of global DNA methylation. In various cancers such as colon and ovarian cancer, it is thought that hypomethylation of LINE-1 is correlated with poor prognosis [17], [18], [19]. However, in glioma patients, the level of LINE-1 methylation has not been fully estimated. Recently, many studies have suggested that low-grade gliomas (LGGs, WHO grade 2) including astrocytoma (As), oligodendroglioma (OG) and oligoastrocytoma (OA) display a highly methylated profile, in particular LGGs with mutated *IDH1* [20], [21].

In the current study, we aimed to determine the correlation between clinical, genetic, and epigenetic profiles of LINE-1 and of different cancer-related genes and the clinical outcome in newly diagnosed LGG and GBM patients. Here, we demonstrated that (1) LINE-1 methylation levels in primary and secondary GBMs are lower than those in LGGs and normal brain tissues, (2) LINE-1 methylation is directly proportional to *MGMT* promoter methylation in gliomas, and (3) higher LINE-1 methylation is a favorable prognostic factor in primary GBMs. As a global DNA methylation marker, LINE-1 may be a promising marker reflecting the *MGMT* promoter methylation and the G-CIMP status.

## Materials and Methods

### Ethics Statement

The study was approved by the institutional review board at each participating hospital and complied with all provisions of the Declaration of Helsinki.

### Patients and Tumor Samples

We collected 111 freshly frozen tissues from patients with LGGs (WHO grade 2), or GBMs treated at Nagoya University Hospital, Oita University Hospital, Hamamatsu University Hospital, and Shizuoka Cancer Center. Their clinical characteristics are summarized in Table 1. Of 57 LGG patients, 30 patients with residual tumor evaluated by T2-wighted magnetic resonance imaging (MRI) received adjuvant nitrosourea-based or TMZ-based chemotherapy concomitant with radiotherapy (large focal 40 Gy) immediately after initial surgery. All primary GBM patients received TMZ–based chemotherapy and radiotherapy (60 Gy) following initial surgery. Secondary GBM was defined as a prior histological diagnosis of LGG.

**Table 1:** Clinical characteristics.

Baseline characteristics of all gliomas					
(n = 111)					
Histological subgroups	No.	Age, years		Sex	
		median	range	male (%)	female (%)
Grade 2 gliomas	57	42.0	21–72	36 (63%)	21 (37%)
As	17	40.0	23–72	13 (76%)	4 (24%)
OG	29	44.0	21–61	17 (59%)	12 (41%)
OA	11	48.0	26–68	6 (55%)	5 (45%)
GBMs	54	59.0	12–84	33 (61%)	21 (39%)
pGBMs	51	59.2	12–84	31 (61%)	20 (39%)
sGBMs	3	42.0	21–50	2 (67%)	1 (33%)

As; Astrocytoma, OG; Oligodendroglioma, OA; Oligo-astrocytoma, pGBMs; primary GBMs, sGBMs; secondary GBMs.

### Tumor Samples

DNA was prepared using the QIAmp DNA Mini kit (Qiagen, Hilden, Germany) according to the manufacturer's instructions. The amount of DNA obtained from the tumor was sufficient for the subsequent genomic and epigenomic analyses.

### Multiplex Ligation-Dependent Probe Amplification

Multiplex ligation-dependent probe amplification (MLPA) was used to determine allelic losses and gains in the tumor samples. The analysis was performed using the SALSA MLPA KIT P088-B1 and P105-C1 in accordance with the manufacturer's protocol (MRC Holland, Amsterdam, Netherland) [22]. Information regarding the probe sequences and ligation sites can be found at www.mlpa.com. Amplification products were separated on an ABI® 3130×I Genetic Analyzer (Applied Biosystems, Foster City, CA) and quantified with Genemapper 4.0 software (Applied Biosystems). Data analysis was performed with an original Excel-based program based on MRC-Holland's procedures. Normalization for sample data was first performed on control probes, and each tumor sample was then normalized using the data on 2 control samples, using peripheral blood DNA. Single regression for control and tumor data slope correction was performed. Abnormal/normal ratio limits were set at 0.65 and 1.3. Statistical analysis was performed using the same Coffalyser software.

### Pyrosequencing

Tumor DNA was modified with bisulfate using the EpiTect bisulfite kit (Qiagen). Pyrosequencing technology was used to determine the methylation status of the CpG island region of the *MGMT* promoter and LINE-1, as described previously [18], [23]. We used the touchdown PCR method for the *MGMT* promoter and the conventional PCR method for LINE-1. The primer sequences used were the *MGMT* forward primer (5′-TTGGTAAATTAAGGTATAGAGTTTT-3′), the *MGMT* biotinylated reverse primer (5′-AAACAATCTACGCATCCT-3′), the LINE-1 forward primer, (5′-TTTTGAGTTAGGTGTGGGATATA-3′), and the biotinylated reverse primer (5′-AAAATCAAAAAATTCCCTTTC-3′). PCR for the *MGMT* promoter included a denaturation step at 95°C for 30 s, followed by annealing at various temperatures for 45 s, and extension at 72°C for 45 s. PCR for LINE-1 included a denaturation step at 95°C for 30 s, annealing at 50°C for 60 s, and extension at 72°C for 45 s. After PCR, the biotinylated PCR product was purified as recommended by the manufacturer. In brief, the PCR product was bound to streptavidin sepharose HP (Amersham Biosciences, Uppsala, Sweden), and the sepharose beads containing the immobilized PCR product were purified, washed, and denatured using a 0.2 N NaOH solution, and then washed again. Next, 0.3 mM pyrosequencing primer was annealed to the purified single-stranded PCR product, and pyrosequencing was performed using the PSQ HS 96 Pyrosequencing System (Pyrosequencing, Westborough, MA). The pyrosequencing primer for the *MGMT* promoter was 5′–GGAAGTTGGGAAGG-3′ and for LINE-1 was 5′–AGTTAGGTGTGGGATATAGT-3′. Methylation was quantified using the provided software.

### TP53 and IDH1/IDH2 Sequencing

Direct sequencing of *TP53* exons 5 to 8 and *IDH1/2* was performed as previously described [24], [25]. The primer sequences are listed in Table 2. For *IDH* sequencing, 2 fragments were amplified: (1) a 129-bp fragment spanning the sequence encoding the catalytic domain of *IDH1*, including codon 132 and (2) a 150-bp fragment spanning the sequence encoding the catalytic domain of *IDH2*, including codon 172. For sequencing *TP53*, we applied touchdown PCR using the standard buffer conditions; the reaction mixture included 5 ng of DNA and AmpliTaq Gold DNA Polymerase (Applied Biosystems). The reaction was run for 16 cycles with denaturation at 95°C for 30 s, annealing at 65–57°C (decreasing by 0.5°C per cycle) for 30 s, and extension at 72°C for 60 s, in a total volume of 12.5 ml. Then, an additional 30 cycles were performed with denaturation at 95°C for 30 s, annealing at 55°C for 30 s, and extension at 72°C for 60 s, ending at 72°C for 7 min to complete extension. Direct sequencing was performed using the BigDye Terminator v1.1 Cycle Sequencing Kit (Applied Biosystems). The reactions were carried out using an ABI 3100 Genetic Analyzer (Applied Biosystems). For *IDH1/2* mutations, we applied conventional PCR at 35 cycles with denaturation at 95°C for 30 s, annealing at 56°C for 40 s, and extension at 72°C for 50 s, ending at 72°C for 7 min to complete extension.

**Table 2:** List of Primer Sequences for Direct DNA Sequencing.

Gene name	Exon	Sequence
*TP53*	Exon 5	F 5′-TTATCTGTTCACTTGTGCCC-3′
		R 5′-ACCCTGGGCAACCAGCCCTG-3′
	Exon 6	F 5′-ACGACAGGGCTGGTTGCCCA-3′
		R 5′-CTCCCAGAGACCCCAGTTGC-3′
	Exon 7	F 5′-GGCCTCATCTTGGGCCTGTG-3′
		R 5′-CAGTGTGCAGGGTGGCAAGT-3′
	Exon 8	F 5′-CTGCCTCTTGCTTCTCTTTT-3′
		R 5′-TCTCCTCCACCGCTTCTTGT-3′
*IDH1*		F 5′-CGGTCTTCAGAGAAGCCATT-3′
		R 5′-GCAAAATCACATTATTGCCAAC-3′
*IDH2*		F 5′-AGCCCATCATCTGCAAAAAC-3′
		R 5′-CTAGGCGAGGAGCTCCAGT-3′

F indicates forward primer, R,reverse primer.

### Statistical Analysis

Statistical analysis was performed using the statistical software SPSS for Windows, version 19.0 (SPSS Inc, Chicago,I ll). The Mann-Whitney U test, the Student's t-test, the χ^2^ test, and the Fisher exact test were used to test for the association of clinical variables and molecular markers. Correlation of methylation level between *MGMT* promoter and LINE-1 was analyzed by using Spearman rank correlation coefficient, and analyzed by using Pearson product - moment correlation coefficient in LGGs. Survival was estimated by using the Kaplan-Meier method, and survival curves were compared by using the log-rank test. Overall survival (OS) was calculated from the day of initial surgery until death or the end of follow-up, and progression-free survival (PFS) was until tumor progression or re-treatment. Among LGGs, univariate and multivariate analyses were performed to test the potential influence of baseline characteristics on OS and PFS. The effect of each single factor on OS and PFS was investigated using the Cox proportional hazards model, adjusting for the major clinical prognostic factors, including age at diagnosis (<40 vs. ≥40), Sex (male vs. female), Eastern Cooperative Oncology Group (ECOG) performance status score (ECOG PS; ≤1 vs. >1), extent of resection (macroscopic [gross] total resection [GTR] or subtotal resection [STR] vs. partial resection or biopsy), *MGMT* promoter methylation status, chromosome *1p* loss of heterozygosity (LOH), *19q* LOH, *PTEN* loss, *CDKN2A* loss, *TP53* loss and mutation, *ERBB2* amplification, *EGFR* amplification, *IDH1* and *IDH2* mutation, and adjuvant therapy immediately following the surgery (with radiotherapy or chemotherapy vs. none). The factors in the multivariate proportional hazard model (p<0.05) were considered independent factors correlated with prolongation of OS and PFS.

## Results

### Frequency of Genetic and Epigenetic Alterations in LGGs, and Primary and Secondary GBMs

**Figure 1:**
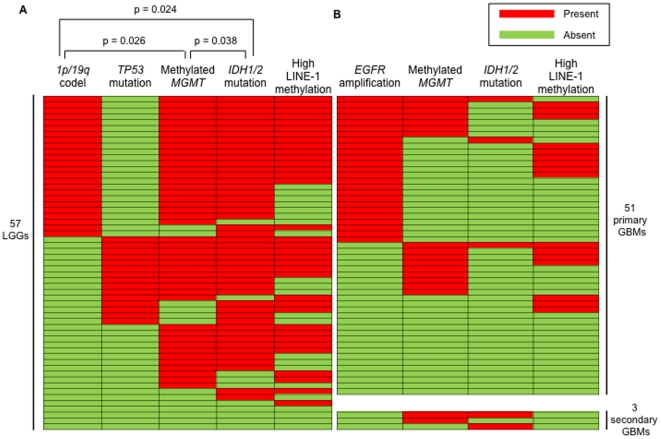
Correlations between the methylation status of the *MGMT* promoter, *IDH1/2* mutations, and *1p/19q* deletions, higher LINE-1 methylation in low-grade gliomas (LGGs), *EGFR* amplification, *MGMT* promoter, *IDH1/2* mutations, high LINE-1 methylation in primary and secondary GBMs. Using the χ^2^ test in grade 2 gliomas, *IDH1/2* mutation was correlated significantly with a methylated *MGMT* promoter (p = 0.038) and *1p/19q* codeletion (p = 0.024). Further, the presence of a methylated *MGMT* promoter was correlated significantly with *1p/19q* codeletion (p = 0.026). Additionally, of the 24 cases with *1p/19q* codeletion, 23 and 22 cases exhibited *IDH1/2* mutations and methylated *MGMT* promoters, respectively, but none showed *TP53* mutations. Of the 44 cases with methylated MGMT promoters, 39 cases exhibited *IDH1/2* mutations (A). In primary and secondary GBMs, *EGFR* amplification, which is the most frequent, and methylated *MGMT* promoter, *IDH1/2* mutation and high LINE-1 methylation was shown (B).

We used direct sequencing for *TP53* and *IDH1/2* and employed MLPA for the analysis of *1p/19q* loss, *PTEN* and *CDKN2A* loss, and amplification of *ERBB2* and *EGFR*. Moreover, we used pyrosequencing technology for quantitative estimation of the methylation status of the *MGMT* promoter and LINE-1. Based on comparisons using standard methylation-specific PCR and immunohistochemical studies using the anti-MGMT antibody, we determined 14% as the threshold distinguishing unmethylation from methylation of the *MGMT* promoter in a given tumor, as reported previously [26]. The data are summarized in Table 3 and Figure 1. In LGGs, *IDH1/2* mutation and methylation of the *MGMT* promoter were frequently observed (∼80%). Of the 46 tumors with *IDH1* mutations, 44 exhibited R132H, one R132G, and one R132S. The *1p/19q* codeletion was detected more often in OG (72%) than in As (6%) and OA (18%). In contrast, *TP53* mutation was more frequently observed in As (41%) and OA (45%) than in OG (10%). We did not detect amplification of *EGFR and ERBB2* in LGGs. In comparison with primary GBM, secondary GBM had more *IDH1/2* and *TP53* mutations and *CDKN2A* loss, a higher frequency of methylated *MGMT* promoter, and less *EGFR* amplification, although the number of secondary GBM (n = 3) was limited (Table 3, Figure 1B).

**Table 3:** Genetic, Epigenetic Alterations in all gliomas.

	Grade 2 gliomas			GBMs		Total
	As	OG	OA	pGBMs*	sGBMs	
	n = 17	n = 29	n = 11	n = 51	n = 3	n = 111
*IDH1/2* mutation	14 (82%)	24 (83%)	9 (82%)	3 (6%)	2 (67%)	52 (47%)
*TP53* mutation	7 (41%)	3 (10%)	5 (45%)	19 (37%)	3 (100%)	37 (33%)
*1p* LOH	1 (6%)	21 (72%)	2 (18%)	4 (8%)	1 (33%)	29 (26%)
*19q* LOH	7 (41%)	22 (76%)	4 (36%)	5 (10%)	1 (33%)	39 (35%)
*1p/19q* codeletion	1 (6%)	21 (72%)	2 (18%)	4 (8%)	0	28 (25%)
*PTEN* loss	2 (12%)	0	0	4 (8%)	0	6 (5%)
*CDKN2A* loss	1 (6%)	1 (3%)	1 (9%)	20 (39%)	3 (100%)	26 (23%)
*ERBB2* amplification	0	0	0	1 (2%)	0	1 (1%)
*EGFR* amplification	0	0	0	25 (49%)	0	25 (23%)
Methylated *MGMT*	12 (71%)	24 (83%)	8 (73%)	16 (31%)	2 (67%)	62 (56%)
LINE-1 methylation**	67.6±3.0	69.0±2.6	70.0±2.3	66.6±4.1	60.7±1.8	67.6±3.6

As; Astrocytoma, OG; Oligodendroglioma, OA; Oligo-astrocytoma, pGBMs; primary GBMs, sGBMs; secondary GBMs.

*Motomura K et al reported these alterations of primary GBMs previously [26].

**LINE-1 methylation indicates mean methylation level ± S.D. (%).

Recently, emerging evidence revealed correlations between the methylation status of the *MGMT* promoter, *IDH1* mutations, and *1p/19q* codeletions [27], [28], [29], [30]. Using the χ^2^ test in LGGs, *IDH1/2* mutation was correlated significantly with a methylated *MGMT* promoter (p = 0.038) and *1p/19q* codeletion (p = 0.024). Further, the presence of a methylated *MGMT* promoter was correlated significantly with *1p/19q* codeletion (p = 0.026). Additionally, of the 24 cases with *1p/19q* codeletion, 23 and 22 cases exhibited *IDH1/2* mutations and methylated *MGMT* promoters, respectively, but none showed *TP53* mutations. Of the 44 cases with methylated *MGMT* promoters, 39 cases exhibited *IDH1/2* mutations. These results suggest that almost all patients having tumors with *1p/19q* codeletions exhibited methylated *MGMT* promoters and that almost all tumors with methylated *MGMT* promoters exhibited *IDH1/2* mutations (Figure 1A).

### LINE-1 Methylation Is Proportional to MGMT Promoter Methylation in gliomas

**Figure 2:**
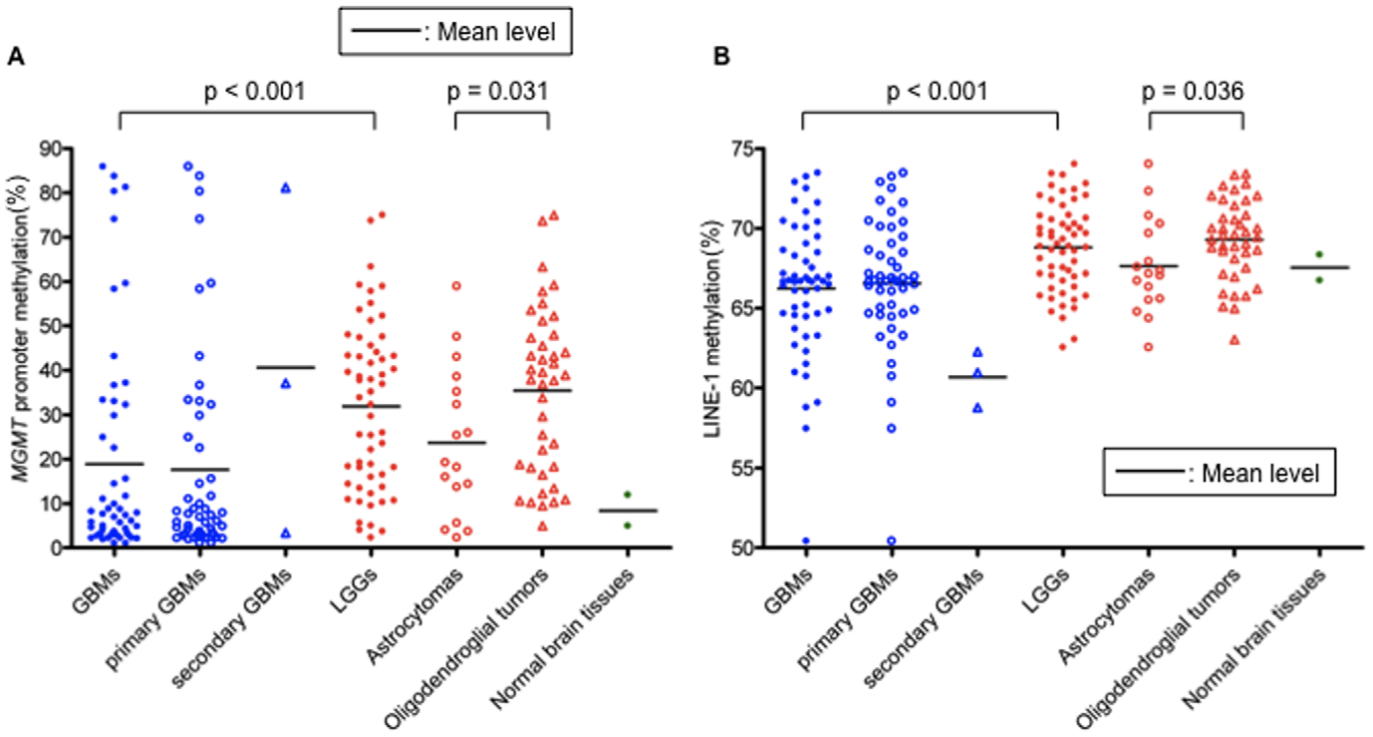
Differences in the methylation levels of *MGMT* promoter and LINE-1 between low-grade gliomas (LGGs) and glioblastoma multiforme (GBM), and between grade 2 astrocytomas and oligodendroglial tumors. A higher proportion of LGGs including astrocytoma, oligodendroglioma, and oligoastrocytoma, exhibited a methylated *MGMT* promoter (A) and LINE-1 (B) compared to GBMs, although the level of LINE-1 in GBMs varied (see also Table 3). Compared among histological subgroups, the level of LINE–1 methylation in astrocytomas was significantly lower than that in oligodendroglial tumors (B), which was similar to the *MGMT* promoter methylation (A). Horizontal line in the graph indicated the mean level.

The level of LINE–1 methylation is regarded as a surrogate of global DNA methylation. Recently, many studies have suggested that low-grade gliomas (LGGs, WHO grade 2) including astrocytoma (As), oligodendroglioma (OG) and oligoastrocytoma (OA) display a highly methylated profile [20], [21]. We examined the level of LINE-1 methylation in comparison with that of *MGMT* promoter methylation in glioma patients. To date, studies have revealed that the level of methylated *MGMT* promoters among LGGs was higher than that among GBMs [20], [30], [31]. Similar to the previous reports, a higher proportion of LGGs exhibited a methylated *MGMT* promoter and LINE-1 compared to GBMs, although the level of LINE-1 in GBMs varied [*MGMT*, mean 18.9% vs. 31.9% (p<0.001); LINE-1, 66.2% vs. 68.8% (p<0.001); Table 3 and Figure 2AB]. Compared among histological subgroups, the level of LINE–1 methylation in As was significantly lower than that in oligodendroglial tumors, including OG and OA, which was similar to the *MGMT* promoter methylation (mean LINE-1 methylation level, 67.6% vs. 69.3%; p = 0.036, Figure 2B).

**Figure 3:**
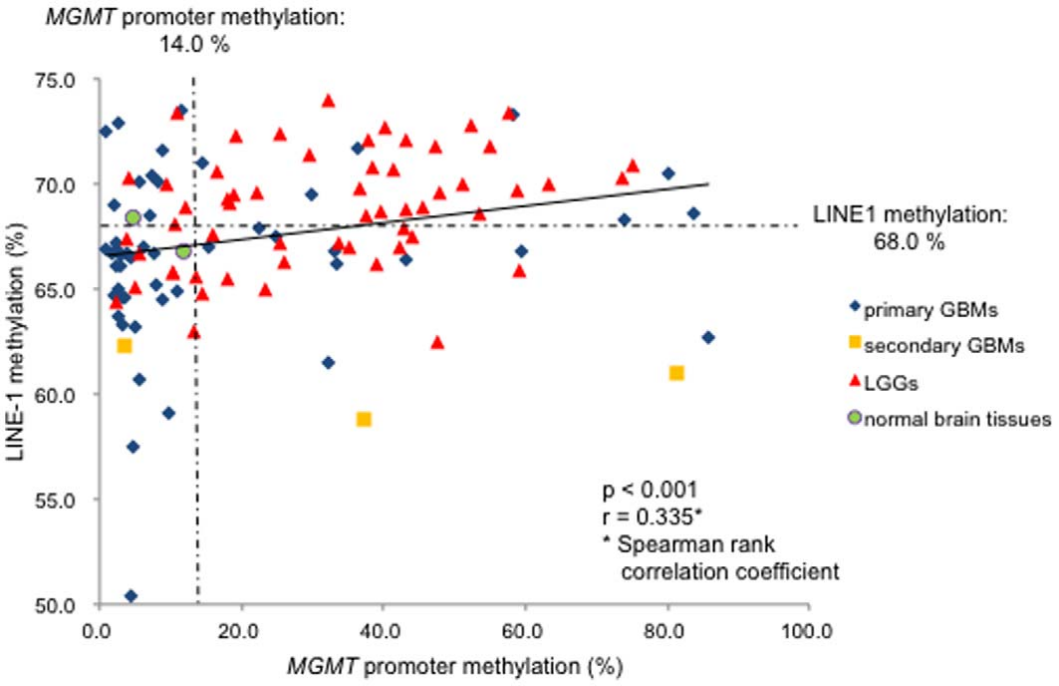
LINE-1 methylation is directly proportional to *MGMT* promoter methylation in gliomas. *MGMT* promoter methylation level was directly proportional to LINE–1 methylation in a statistically significant manner (r = 0.335, p<0.001) for all samples quantified, including LGGs, primary and secondary GBMs, and normal brain tissue. Cut-off line of LINE-1 methylation, *MGMT* promoter methylation was indicated.

The results described above prompted us to analyze the correlation between the quantitative methylation values of LINE-1 and the *MGMT* promoter. We found that the *MGMT* promoter methylation level was directly proportional to LINE–1 methylation in a statistically significant manner (r = 0.335, p<0.001) for all glioma samples and normal brain tissue (Figure 3). However, while LINE-1 methylation is significantly proportional to *MGMT* promoter in LGGs (r = 0.336, p = 0.011), statistical significance was not found when primary GBMs only were analyzed, probably due to non-parametric distribution of the *MGMT* promoter methylation level (Figure S1AB).

Previously, it was reported that G-CIMP tumors are more prevalent among LGGs, and are tightly associated with *IDH1* mutation [16]. Thus, it may be interesting to know whether LINE-1 methylation is correlated with *IDH1* mutation in our sample sets. Although we did not observe the significant correlation between *IDH1/2* mutation and higher LINE-1 methylation both among LGGs and GBMs (Figure S2), we showed that LGGs exhibited higher LINE-1 methylation than GBMs did, and oligodendroglial tumors showed higher LINE-1 methylation than astrocytomas (Table 3, Figure 2B), which was consistent with the previous report demonstrating that LGGs, in particular oligodendroglial tumors are characteristics of G-CIMP positive group.

### Clinical, Genetic, and Epigenetic Parameters in Correlation with PFS and OS in Low-grade Glioma Patients

**Figure 4:**
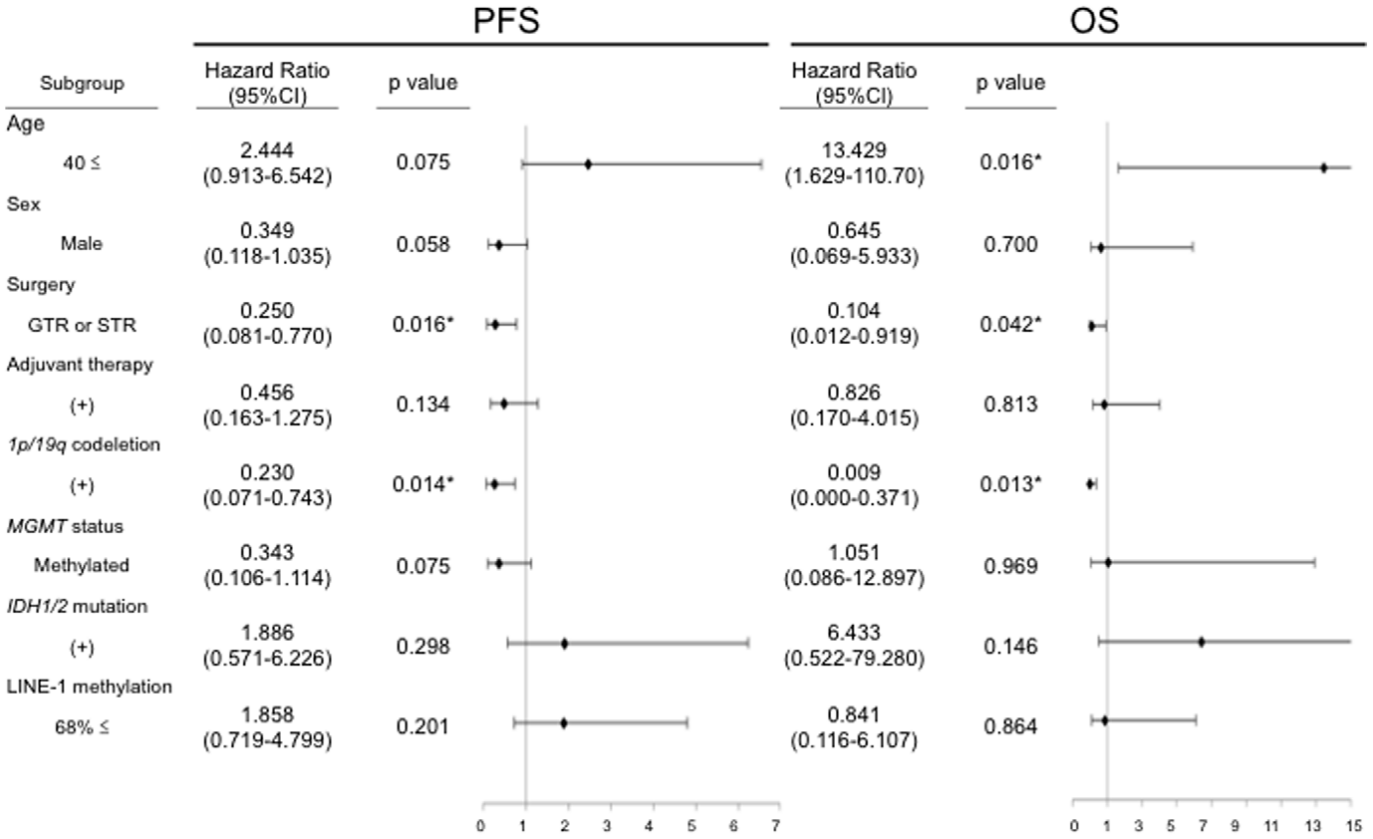
Clinical, genetic, and epigenetic parameters in correlation with progression-free survival (PFS) and overall survival (OS) in low-grade glioma patients. The presence of *1p/19q* codeletion and the extent of resection were independently correlated with prolonged PFS, as shown with multivariate analysis (p = 0.014 and p = 0.016, respectively). The presence of *1p/19q* codeletion, the extent of resection and the age were correlated with prolonged OS (p = 0.013, 0.042, 0.016, respectively).

**Figure 5:**
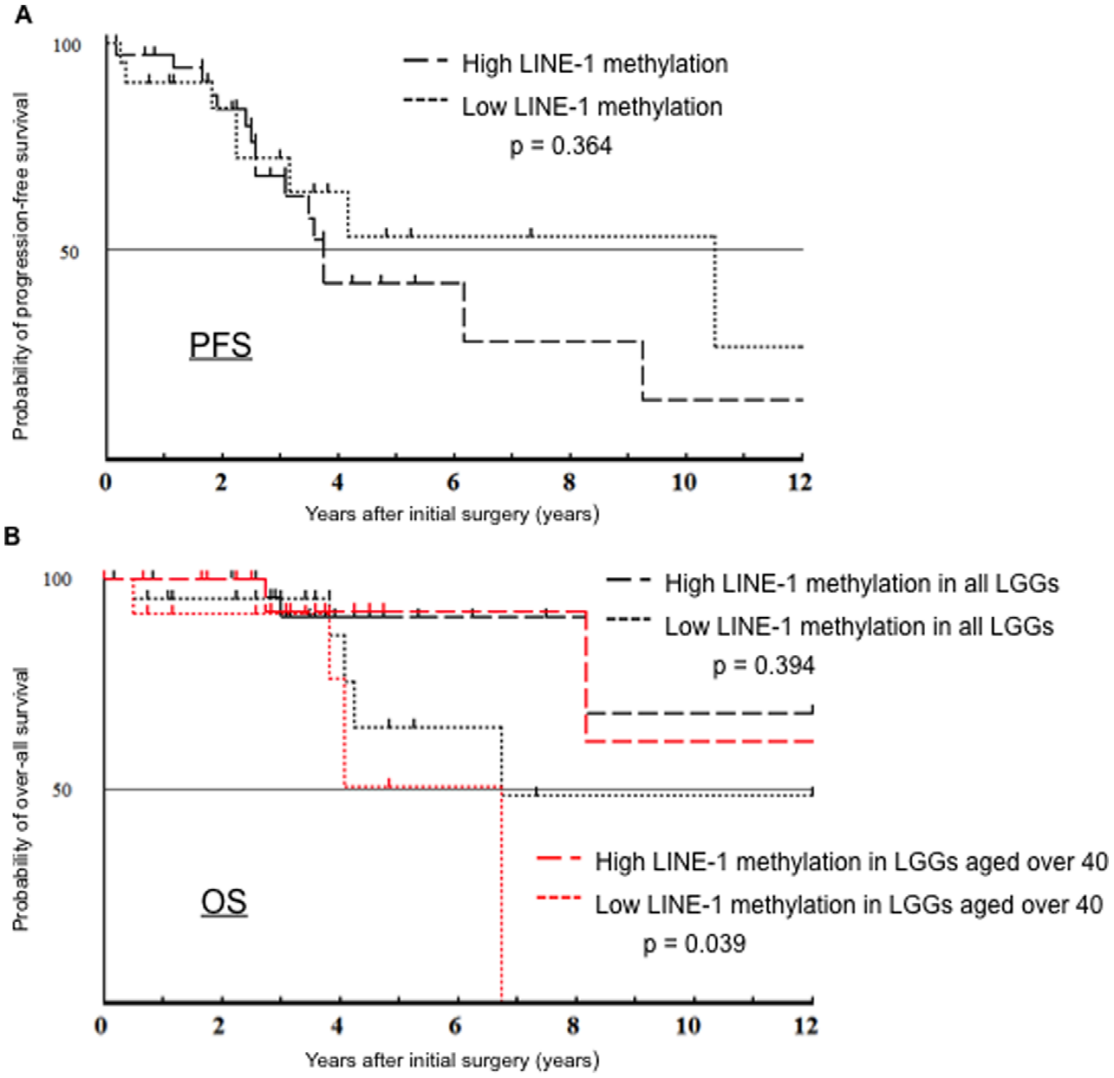
High LINE-1 methylation status in correlation with progression-free survival (PFS) and overall survival (OS) in low-grade glioma patients. In the Kaplan-Meier survival curve of patients with LGGs, High LINE-1 methylation status was not correlated with PFS in LGGs, using log-rank test (p = 0.364); (A). However in correlation with OS, in LGGs aged over 40, High LINE-1 methylation prolonged OS (p = 0.039), black line indicated the Kaplan-Meier survival curve of all LGGs (high LINE-1 methylation and low), red line LGGs aged over 40 (B).

We investigated the correlations of the genetic and epigenetic alterations with OS and PFS among LGGs. Among all LGGs, the median PFS was 45.7 months (95% confidence interval [CI]: 17.1–74.3 months), the median OS was 172.8 months (95%CI; 8.9–336.8 months). Patients with As, OG, and OA had a PFS of 45.1, 74.9, and 37.3 months, respectively. As shown in Figure 4, the presence of *1p/19q* codeletion, the extent of resection were independently correlated with PFS, as shown with multivariate analysis (p = 0.014, 0.016), and the presence of *1p/19q* codeletion, the extent of resection and the age were correlated with prolonged OS (p = 0.013, 0.042, 0.016, respectively). Using a log-rank test, a univariate analysis revealed that prolonged PFS and OS was significantly correlated only with the presence of *1p/19q* codeletion (p = 0.013, p = 0.013, supplementary Figure S3AB). Univariate analysis showed that a methylated *MGMT* promoter was not significantly correlated with prolonged PFS (p = 0.128); however, if patients undergoing partial removal or biopsy at initial surgery were selected, it became significantly correlated with PFS (p = 0.017, supplementary Figure S4). Of particular note, high LINE-1 methylation (68% ≤) was significantly correlated with prolonged OS of patients aged over 40 (p = 0.039), whereas statistical significant association was not obtained between high LINE-1 methylation and PFS (Figure 5).

### LINE-1 Methylation is a Prognostic Factor Among primary GBMs

**Figure 6:**
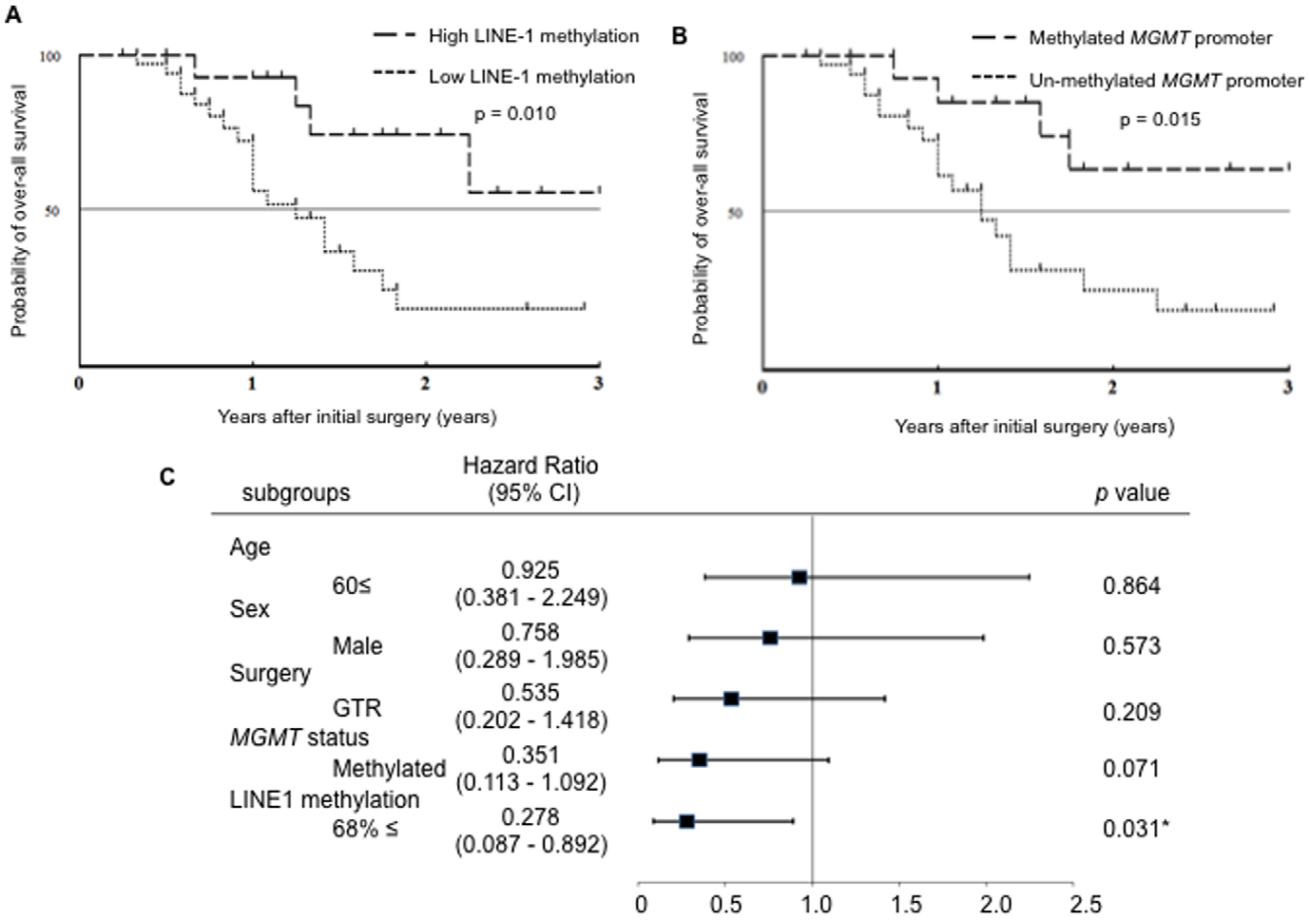
LINE-1 methylation is a better prognostic indicator in primary GBMs. In the Kaplan-Meier survival curve of patients with primary GBM, univariate analysis indicated a lower p value in the comparison of <68% and ≥68% of LINE-1 methylation (A) than in the comparison of <14% and ≥14% of *MGMT* promoter methylation (B). In multivariate analysis, the hazard ratio was computed using a proportional hazard model by selected factors. Prolonged overall survival time was significantly correlated with a high LINE-1 methylation status but not with a methylated *MGMT* promoter (C).

Next, we examined whether LINE-1 could be a prognostic factor in primary GBMs. To our surprise, in the Kaplan-Meier survival curve of patients with primary GBM, univariate analysis indicated a lower p value in the comparison of <68% and ≥68% of LINE-1 methylation than in the comparison of <14% and ≥14% of *MGMT* promoter methylation (p = 0.010 and 0.015, Figure 6AB). Furthermore, in multivariate analysis, the hazard ratio was computed using a proportional hazard model by selected factors. Prolonged overall survival time was significantly correlated with a high LINE-1 methylation status but not with a methylated *MGMT* promoter (p = 0.031, Figure 6C).

### Genetic and Epigenetic Changes From Low-grade Glioma to Secondary GBM

We experienced 3 secondary GBM cases and obtained serial tumor samples of 2 cases at the time of grade 2 glioma (As and OA) and at the time of progression to GBM. The secondary GBM tumors already had *TP53* mutation and *IDH1* mutation at the time of the low-grade tumors but displayed a 2-fold increase in methylation of the *MGMT* promoter and an 8% decrease in methylation of LINE-1 during malignant transformation.

## Discussion

Previously, we demonstrated clinical, genetic, and epigenetic profiles in newly diagnosed primary GBMs [26]. In this study, we extended those analyses to LGGs, in comparison with GBMs. We also included secondary GBMs in order to provide a possible clue into the profile changes that occur during malignant transformation. Of great interest, the principal and novel finding of the current study is that a global DNA methylation surrogate, LINE-1 methylation, is positively proportional to the *MGMT* promoter methylation in gliomas.

In this study, 57 LGG samples exhibited *IDH1/2* mutations most frequently (82%), followed by methylated *MGMT* promoters (77%), *1p/19q* codeletion (42%), and *TP53* mutations (26%). Our results were consistent with data reported previously [20], [32], [33], [34], [35]. We demonstrated that higher methylation levels of LINE-1 and the *MGMT* promoter and *1p/19q* codeletion were associated with oligodendroglial tumors. Additionally, the presence of *1p/19q* codeletion was significantly correlated with higher *MGMT* promoter methylation.

Of these alterations, *1p/19q* codeletion was most strongly correlated with prolonged OS and PFS in both univariate and multivariate analysis of LGGs. In our study, *IDH1/2* mutation was not correlated with prolonged PFS and OS in LGG patients. The finding was consistent with previous reports demonstrating that *IDH1/2* mutations are not a prognostic factor for LGGs [27], [36], but there was opposed evidence showing significant and independent associations between *IDH* mutation and improved survival in LGGs [21], [32]. The prognostic significance of *IDH1/2* mutation in LGGs remains controversial.

To date, *MGMT* promoter methylation has been regarded as a prognostic as well as predictive for the outcome to adjuvant chemotherapy [10]. In various cancers, such as colorectal cancer, global DNA hypomethylation was correlated with poor prognosis [17], [18]. We hypothesized that *MGMT* promoter hypermethylation reflects global DNA hypermethylation in gliomas. To demonstrate our hypothesis, we quantified the level of LINE-1 methylation in gliomas. Higher methylation levels of LINE-1 and the *MGMT* promoter were observed in LGGs than in GBMs (LINE-1: mean 68.8% vs. 66.2%, p<0.001; *MGMT* promoters: 31.9% vs. 18.9%, p<0.001). Additionally, we investigated the correlations between LINE-1 and *MGMT* promoter methylation levels. Among gliomas, in particular LGGs, LINE-1 methylation levels were significantly proportional to *MGMT* promoter methylation. Notably, only low LINE-1 methylation indicated poor prognosis in primary GBM patients, as analyzed by both univariate and multivariate analyses. Prolonged overall survival time was significantly correlated with high LINE-1 methylation status but not with a methylated *MGMT* promoter. Additionally, higher LINE-1 methylation was correlated with prolonged OS in LGG patients aged over 40. This is consistent with other cancers such as colorectal cancer and ovarian cancer, in which hypomethylation of LINE-1 is correlated with shortened survival [17], [18], [37].

LINE-1 methylation and *MGMT* promoter methylation were also correlated with tumor grading; LGGs displayed a higher methylation level of LINE-1 and the *MGMT* promoter than GBMs (WHO grade 4). Thus, in order to determine whether DNA methylation relies on malignant transformation, we investigated changes in genetic and DNA methylation patterns from LGGs to secondary GBMs in identical cases. However, secondary GBMs paradoxically displayed an increase in *MGMT* promoter methylation and a decrease in LINE-1 methylation. The limited number of samples studied warrant further investigations.

Previously, it was reported that G-CIMP tumors are tightly associated with *IDH1* mutation [16]. More recently, *IDH* mutations and resultant 2-hydroxyglutarate (2HG) production in leukemia cells were reported to induce global DNA hypermethylation through impaired TET2 catalytic function [38]. In this study, LGGs with *IDH1/2* mutation tended to exhibit higher LINE-1 methylation although there was no statistical significance. Our study demonstrated the correlation of LINE-1 methylation with good prognosis among GBMs for the first time, however, the mechanism was not interpreted and the number of samples in our study was limited. The higher levels of LINE-1 methylation in low grade gliomas may be attributable to the differential prevalence of *IDH* mutation in low versus high-grade glioma, and the methylator phenotype associated with *IDH* mutation. Thus, interpreting LINE-1 methylation values for prognosis may be more difficult than interpreting *IDH1/2* mutation. We need further investigation to validate our findings.

In summary, we demonstrated that LINE-1 methylation levels in primary and secondary GBMs are lower than those in LGGs and normal brain tissues, that LINE-1 methylation is directly proportional to *MGMT* promoter methylation in gliomas, and that higher LINE-1 methylation is a favorable prognostic factor in primary GBMs. LINE-1 is a global DNA methylation marker, which may be a promising marker reflecting the *MGMT* promoter or the G-CIMP status.

## Supporting Information

Figure S1 **Correlation between the methylation levels of LINE-1 and** ***MGMT*** **promoter.** Among LGGs, LINE-1 is derectly proportional to *MGMT* promoter, p = 0.011, r = 0.336 (A). However among primary GBMs, the correlation between the methylation levels of LINE-1 and *MGMT* promoter are statistically insignificant, p = 0.187, r = 0.188 (B).
(TIFF)

Figure S2 **Differences of methylation levels of LINE-1 between mutated** ***IDH1/2*** **and wild-type.** Among LGGs, *IDH1/2* mutation exhibited higher methylation level of LINE-1, although insignificant, than wild-type *IDH1/2*, mean; 69.0±2.5%, 67.6±3.4%, p = 0.144 (A). Among primary and secondary GBMs, mutated *IDH1/2* did not exhibited the differences of methylation level of LINE-1, compared with wild-type *IDH1/2* although we analyzed only 5 mutated *IDH1/2*, mean; 65.5±4.8%, 66.3±4.2%, p = 0.449 (B).
(TIFF)

Figure S3 ***1p/19q*** **codeletions in correlation with over-all survival, progression-free survival in low-grade glioma patients.** Using a log-rank test, a univariate analysis revealed that prolonged PFS (A) and OS (B) was significantly correlated only with the presence of *1p/19q* codeletion.
(TIFF)

Figure S4 ***MGMT*** **promoter methylation in correlation with progression-free survival (PFS) in low-grade glioma patients.** Methylated *MGMT* promoter was not significantly correlated with prolonged PFS (A); however, if patients undergoing partial removal or biopsy at initial surgery were selected, it became significantly correlated with PFS (B).
(TIFF)
