# Supplementary figures and images for: The Global DNA Methylation Surrogate LINE-1 Methylation Is Correlated with MGMT Promoter Methylation and Is a Better Prognostic Factor for Glioma
Source: PLoS One. 2011 Aug 4;6(8):e23332. doi: 10.1371/journal.pone.0023332 (PMC3150434; doi:10.1371/journal.pone.0023332)

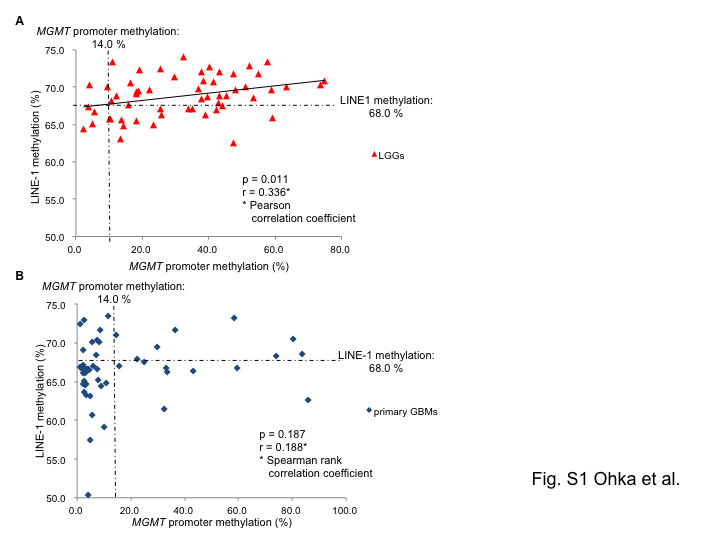

Supplement: Figure S1 — Correlation between the methylation levels of LINE-1 and MGMT promoter. Among LGGs, LINE-1 is derectly proportional to MGMT promoter, p = 0.011, r = 0.336 (A). However among primary GBMs, the correlation between the methylation levels of LINE-1 and MGMT promoter are statistically insignificant, p = 0.187, r = 0.188 (B). (TIFF) [file pone.0023332.s001.tiff]

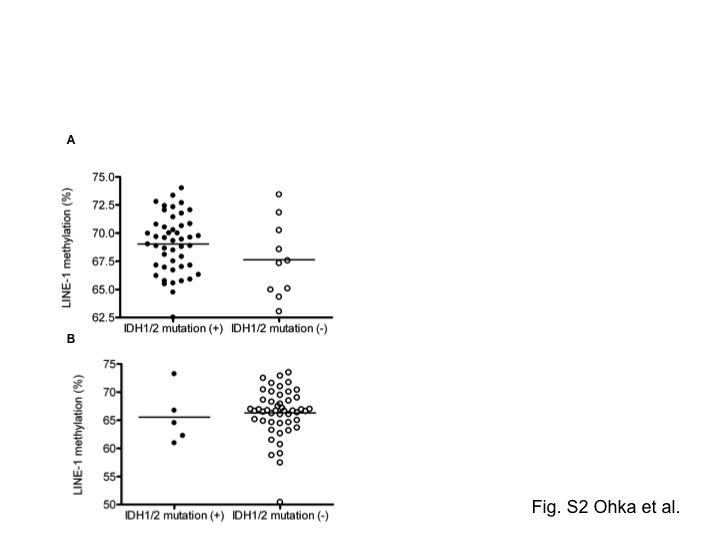

Supplement: Figure S2 — Differences of methylation levels of LINE-1 between mutated IDH1/2 and wild-type. Among LGGs, IDH1/2 mutation exhibited higher methylation level of LINE-1, although insignificant, than wild-type IDH1/2, mean; 69.0±2.5%, 67.6±3.4%, p = 0.144 (A). Among primary and secondary GBMs, mutated IDH1/2 did not exhibited the differences of methylation level of LINE-1, compared with wild-type IDH1/2 although we analyzed only 5 mutated IDH1/2, mean; 65.5±4.8%, 66.3±4.2%, p = 0.449 (B). (TIFF) [file pone.0023332.s002.tiff]

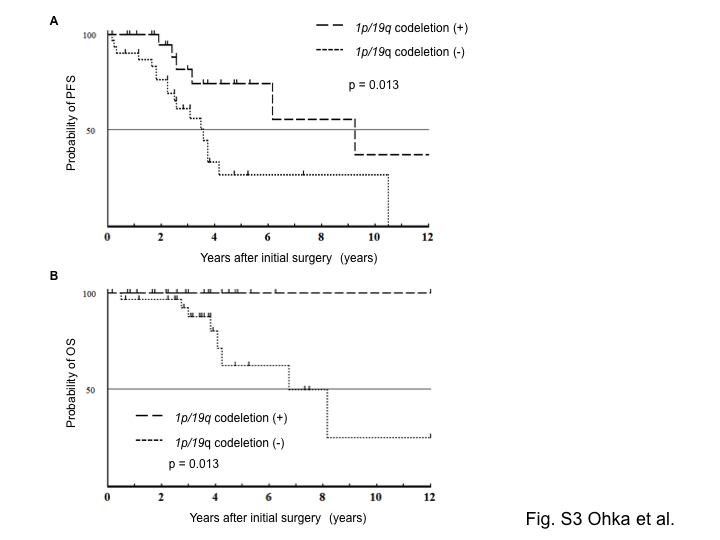

Supplement: Figure S3 — 1p/19q codeletions in correlation with over-all survival, progression-free survival in low-grade glioma patients. Using a log-rank test, a univariate analysis revealed that prolonged PFS (A) and OS (B) was significantly correlated only with the presence of 1p/19q codeletion. (TIFF) [file pone.0023332.s003.tiff]

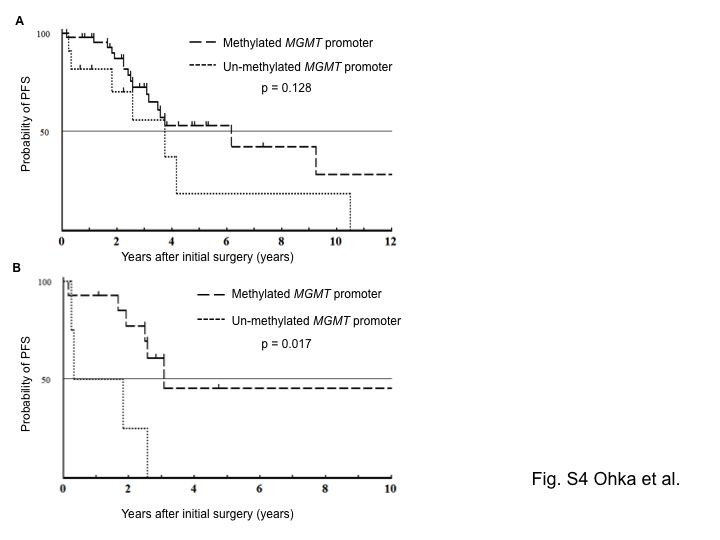

Supplement: Figure S4 — MGMT promoter methylation in correlation with progression-free survival (PFS) in low-grade glioma patients. Methylated MGMT promoter was not significantly correlated with prolonged PFS (A); however, if patients undergoing partial removal or biopsy at initial surgery were selected, it became significantly correlated with PFS (B). (TIFF) [file pone.0023332.s004.tiff]
